# MyCTC chip: microfluidic-based drug screen with patient-derived tumour cells from liquid biopsies

**DOI:** 10.1038/s41378-022-00467-y

**Published:** 2022-12-20

**Authors:** Fabienne D. Schwab, Manuel C. Scheidmann, Lauren L. Ozimski, André Kling, Lucas Armbrecht, Till Ryser, Ilona Krol, Karin Strittmatter, Bich Doan Nguyen-Sträuli, Francis Jacob, André Fedier, Viola Heinzelmann-Schwarz, Andreas Wicki, Petra S. Dittrich, Nicola Aceto

**Affiliations:** 1 Department of Biomedicine, Cancer Metastasis Laboratory, University of Basel, Basel, Switzerland; 2 Department of Gynaecologic Oncology, University Hospital Basel, Basel, Switzerland; 3 Department of Biology, Swiss Federal Institute of Technology Zurich (ETH Zurich), Zurich, Switzerland; 4 Department of Biosystems Science and Engineering, Swiss Federal Institute of Technology Zurich (ETH Zurich), Basel, Switzerland; 5 Department of Gynaecology, University Hospital Zurich and University of Zurich, Zurich, Switzerland; 6 Department of Biomedicine, Ovarian Cancer Research, University Hospital Basel and University of Basel, Basel, Switzerland; 7 University of Zurich and University Hospital Zurich, Zurich, Switzerland

**Keywords:** Engineering, Materials science

## Abstract

Cancer patients with advanced disease are characterized by intrinsic challenges in predicting drug response patterns, often leading to ineffective treatment. Current clinical practice for treatment decision-making is commonly based on primary or secondary tumour biopsies, yet when disease progression accelerates, tissue biopsies are not performed on a regular basis. It is in this context that liquid biopsies may offer a unique window to uncover key vulnerabilities, providing valuable information about previously underappreciated treatment opportunities. Here, we present MyCTC chip, a novel microfluidic device enabling the isolation, culture and drug susceptibility testing of cancer cells derived from liquid biopsies. Cancer cell capture is achieved through a label-free, antigen-agnostic enrichment method, and it is followed by cultivation in dedicated conditions, allowing on-chip expansion of captured cells. Upon growth, cancer cells are then transferred to drug screen chambers located within the same device, where multiple compounds can be tested simultaneously. We demonstrate MyCTC chip performance by means of spike-in experiments with patient-derived breast circulating tumour cells, enabling >95% capture rates, as well as prospective processing of blood from breast cancer patients and ascites fluid from patients with ovarian, tubal and endometrial cancer, where sensitivity to specific chemotherapeutic agents was identified. Together, we provide evidence that MyCTC chip may be used to identify personalized drug response patterns in patients with advanced metastatic disease and with limited treatment opportunities.

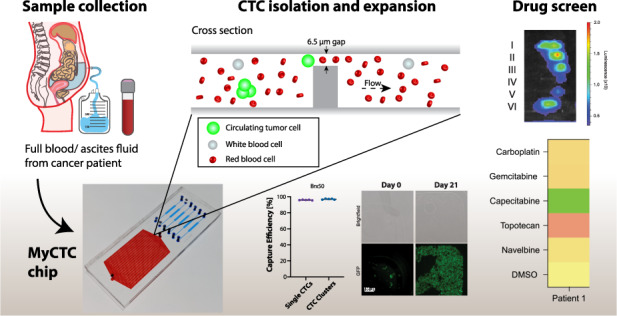

## Introduction

Cancer is one of the leading causes of death worldwide, with 18.1 million new diagnoses and 9.9 million deaths due to cancer each year^1^. Drug-resistant metastases are responsible for the vast majority of cancer-related deaths^2^. These numbers highlight the need for more effective anticancer therapies, including metastasis-tailored targeted treatments. Clinically, widely adopted methods to evaluate the treatment response of solid tumours include quantitative image analysis (e.g., computed tomography (CT), positron emission tomography (PET) or magnetic resonance imaging (MRI))^3^. Additionally, the evaluation of core needle biopsies collected prior to and during treatment provides valuable insights into pharmacodynamics and biomarker changes; however, this method may suffer from inaccuracy owing to sampling of stochastic locations within the tumour^4,5^. In addition, tissue biopsies are invasive, and in an advanced disease stage, some metastases are poorly accessible due to their location, e.g., brain metastasis^6^. Treatment decisions in a metastatic setting are thus mostly based on the characteristics of early biopsies of the primary tumour or metastasis, frequently not reflecting the genomic and phenotypic features of the current tumour^7,8^. First- and second-line therapies are recommended by the European Society of Medical Oncology (ESMO) and National Comprehensive Cancer Network (NCCN), yet there are no clear recommendations for the treatment of advanced cancer beyond third-line therapy^9^.

In contrast to the analysis of needle biopsies, liquid biopsy samples provide a minimally invasive method to assess treatment responses of cancer patients, with potentially higher accuracy^10,11^. The term “liquid biopsies” generally refers to the isolation and interrogation of tumour-derived material such as circulating tumour DNA (ctDNA), extracellular vesicles (EVs) and circulating tumour cells (CTCs) from body fluids of patients (blood and fluid from ascites and pleural effusion) with different types of tumours^12^. CTCs are cancer cells that detach from primary or secondary lesions and are found in the blood of patients with solid cancers^13–15^. In recent decades, multiple methods for CTC isolation have been developed, leading to the clinical assessment of CTCs as a blood-based biomarker, which was approved by the FDA for use in breast, prostate, and colorectal cancer. Clinical trials have shown that higher concentrations of CTCs are associated with worse outcomes and prognoses in these cancers^16–19^. Additional studies assessed the mutational and transcriptional profiles of CTCs and highlighted intra- and interpatient heterogeneity^20–26^, demonstrating the promise of CTCs bearing valuable information to uncover the underlying biology of cancer progression. Along those lines, a case study using genomic profiling of CTCs from non-small cell lung cancer (NSCLC) patients without detectable metastasis could predict the subsequent mutational landscape of metastasis upon relapse^27^. In parallel, malignant abdominal fluid (ascites fluid) frequently develops in patients with advanced high-grade serous and advanced endometrial cancer and is also associated with drug resistance and a poor prognosis^28,29^. Thus, CTCs and cancer cells from ascites fluid hold valuable information about tumour properties that favour metastatic spread, making them a valuable resource for drug susceptibility testing. Although successful cultures of CTCs from breast^30^, colon^31^, lung^32^, ovarian^33^ and prostate^34^ cancer patients have been established, the overall culture efficiency of isolated CTCs remains low (<20%)^35^, presumably owing to the poor viability of CTCs ex vivo and challenges in recapitulating the tumour microenvironment, among other factors^30,36^. Before cultivation, enrichment of viable CTCs from blood specimens is pivotal, and two enrichment strategies can be applied, i.e., antigen-dependent^37–39^ or antigen-independent^40–46^ methods. While antigen-dependent technologies rely on detecting surface antigens of CTCs that are absent in blood cells, such as EpCAM and epithelial cytokeratins, antigen-independent technologies exploit the physical properties of CTCs, such as size and deformability, providing a more unbiased tool for their isolation and enrichment^12^. While the majority of these technologies have been successfully applied mainly for the purpose of CTC enumeration and molecular characterization, fewer technologies allow on-chip CTC expansion and drug screen. To our knowledge, existing devices either include preprocessing steps such as red blood cell (RBC) lysis (which may result in CTC loss) or rely on white blood cell (WBC) coculture systems, which excludes patients with chemotherapy-induced neutropenia^47^. WBC coculture systems can also lead to drug-induced cytotoxicity of WBCs during drug screens, possibly leading to the release of cellular material (e.g., ATP) into the culture environment with an effect on cancer cell viability^35,48,49^. Thus, a microfluidic device allowing simultaneous capture, helper-cell independent culture and drug screening of unprocessed patient-derived cancer cells is lacking, and its development may favour personalized medicine approaches and support clinical decision-making.

## Materials and methods

### Blood and ascites fluid samples

All procedures involving blood samples and ascites fluid samples from patients were performed upon signed informed consent of the participants. Procedures were carried out according to protocols KEK BASEC 2021-01939, EKNZ BASEC 2020-00014 and EKNZ BASEC 2017-01900, approved by the ethical and institutional review board (Ethics Commission Kanton Zurich [KEK] and Ethics Committee Northwest and Central Switzerland [EKNZ]), and in compliance with the Declaration of Helsinki.

### Microfluidic chip fabrication

Fabrication of the “My Circulating Tumour Cell Chip” (MyCTC chip) is based on the following procedure. A negative two-layer master mould of the microfluidic channels is prepared by standard SU-8 photoresist lithography on a 4” silicon wafer substrate. The first layer, defining the capturing gap width, is prepared by spin coating SU-8 3005 (Kayaku Advanced Material Inc., USA) at 2250 rpm for 30 s, resulting in a height of 6.5 µm over a total length of 39.7 cm. Subsequently, the wafer is soft baked and exposed to UV light through a foil mask according to the manufacturer’s recommendations. A second layer of SU-8 3025 is spin coated at 2500 rpm for 30 s, soft baked and exposed to UV light, resulting in a height of an additional 40 µm. After a final postexposure bake, the wafer is developed in a developer bath and hard baked. This initial SU-8 structure serves as a master mould for the “imprinting stamp” that transfers the pattern onto the cyclic olefin copolymer (COC) thermoplastic material by thermal imprinting. The imprinting stamp is prepared by transfer of the initial structure to the temperature- and pressure-resistant UV-curable resist Ormostamp (Micro Resist, Germany) on a glass wafer substrate. After silanization of the stamp with trichloro(1*H*,1*H*,2*H*,2*H*-perfluorooctyl)silane (PFOTS), a compact nanoimprinting tool (CNIV2.0, NILT, Denmark) is used to imprint a COC foil (COC 8007 foil, 240 µm thick, microfluidic ChipShop, Germany) with microchannels. The microchannels are imprinted with a pressure of 6 bar for 4 min at 130 °C. The imprinted COC foils are sealed with a 5 mm thick polydimethylsiloxane (PDMS) slab with punched inlet and outlet holes. PDMS is prepared by mixing the curing agent and base polymer at a 1:10 ratio. Subsequently, the mixture is degassed and cured at 80 °C for at least 3 days in an oven. After dicing, the COC and PDMS pieces are treated with a 5% (3-aminopropyl)triethoxysilane (APTES) solution and a 5% (3-glycidyloxypropyl)trimethoxysilane (GPTMS) solution in water, respectively, for 15 min at 40 °C, as depicted in Fig. S1. Subsequently, the individual pieces are rinsed with DI water and dried with nitrogen. Finally, the channel layer and the sealing layer are aligned and brought into contact. After a hard bake at 50 °C for 1 h, a strong covalent bond between the layers is achieved. Before use, the capture chamber is coated with an anti-adherence rinsing solution (StemCell Technologies) and incubated for 1 h at room temperature to create an ultralow attachment surface, as nonadherent culture conditions are critical to avoid senescence of CTCs. Subsequently, the culture chamber was washed with 3 mL PBS.

### Culturing CTC-derived cell lines

GFP- or RFP-tagged human CTC-derived cell lines were cultured under hypoxic conditions (5% O_2_) in ultralow attachment (ULA) 6-well plates (Corning, 3471-COR). Every third day, CTC cultures were given CTC growth medium, which was made of RPMI 1640 medium (Invitrogen, 52400-025) containing 20 ng mL^−1^ recombinant human epidermal growth factor (Gibco, PHG0313), 20 ng mL^−1^ recombinant human fibroblast growth factor (Gibco, 100-18B), 1X B27 supplement (Invitrogen, 17504-044) and 1X antibiotic-antimycotic (Invitrogen, 15240062).

### Simulations of shear forces within the capture and culture chamber

To determine the pressure distribution within the chip, we simulated the flow of blood in the device using COMSOL Multiphysics 5.1. The flow was modelled as laminar with a fixed volumetric flow rate at the inlet and a zero-pressure boundary condition at the outlet of the chip. For determination of shear forces and pressure drops, the fluid’s dynamic viscosity was set to 1–3 mPa∙s, which resembles the properties of whole blood at room temperature. The operating flow rate was set to 50 µL min^−1^.

### Determination of capture efficiency

To determine the capture efficiency, 100–500 cells of the CTC-derived cell lines (GFP- or RFP-tagged) were spiked into 1 mL healthy donor blood (EDTA, Blutspendezentrum SRK beider Basel, 99970), which was collected in EDTA blood collection tubes (Vacuette, Cat# 455036). Prior to applying the spiked cells to the MyCTC chip, the chip was filled with PBS, and the air was removed by centrifuging the chip at 1000 x g for 10 min. The blood cell mixture was then applied to the capture and culture section with a flow rate of 50 µL min^−1^ using a syringe pump (neMESYS, Cetoni). The outlet was connected to a well of a 24-well ULA plate (Corning, Cat# 3473) via PTFE tubing (1/16'' OD) for subsequent quantification of cells that passed the filter section. After the blood cell mixture was applied, the capture and culture chamber was washed with 1 mL 1X PBS (Gibco, Cat# 14190-094), and GFP- or RFP-positive single and clustered cells captured on the chip were enumerated. The capture efficiency was calculated from the number of CTCs found in the waste and the number of CTCs captured on-chip.

### Determination of release efficiency

The release of captured CTCs from the capture and culture section was performed by injecting 1 mL PBS in the opposite direction than used for the capture, with a flow rate of 30 µL s^−1^. The outlet was connected to a well of a 6-well ULA plate via PTFE tubing. After release, GFP- and RFP-positive cells were enumerated on the chip. The release efficiency was calculated by the number of released CTCs found in the well plate divided by the number of captured CTCs that remained in the microchannel and presented as a percentage.

### Determination of translocation efficiency

Single CTCs and CTC clusters were enumerated in a well and then translocated to the different drug screening channels. The translocation of the CTCs into the six drug channels was performed by aspirating the same volume of the released fluid with cells into a pipette tip, which was then put into the individual inlets. The outlets were connected to a 3 mL syringe mounted on a syringe pump (Nemesys, Cetoni) in withdrawal mode, and the cells were again trapped on-chip at a flow rate of 30 µL min^−1^. The translocated CTCs were individually enumerated for each of the six channels, and translocation efficiency was calculated considering the number of translocated cells in all chambers and the total number of cells released.

### Purity and viability assay

To determine the purity and viability of the different CTC-derived cell lines (BR16, Brx07 and Brx50) on-chip and postrelease, 100–500 cells (GFP- or nontagged) were spiked into 1 mL healthy donor blood (EDTA, Blutspende SRK Zürich) and applied to the MyCTC chip capture section with a flow rate of 50 µL min^−1^. Once captured, the cells were washed with 1 mL 1x PBS and stained with a cocktail containing Calcein Violet 450 AM Viability Dye (ThermoFisher; 65-0854-39), Propidium Iodide (PI) Red (ThermoFisher; P3566), AF-488 anti-EpCAM (Cell Signalling Technologies; CST#5198), and AF647 anti-CD45 (Biolegend; 304056). The device was then incubated at room temperature for 15 min before a final wash with 1 mL 1x PBS. Subsequently, the wash solution was exchanged with CTC growth medium. The cells were then counted and classified into either EpCAM+ cancer cells (AF488+) or CD45 + immune cells (AF647+) and either live (calcein violet+) or dead (PI+) cells. Once quantified, the cells were released at a flow rate of 30 µL s^−1^ into a 24-well plate. After release, the cells were stained with calcein violet and PI again to detect any additional dead cells due to the release process and were again quantified as described above. Counts of viable and dead cells from each CTC-derived cell line in a 24-well plate format, directly from culture, were obtained to quantify initial viability before processing.

### Clustering assay

To determine if artificial clustering of BR16 cells occurs on-chip, cells were sorted as singlets using a BD FACSAria III sorter. A total of 5000 single cells were sorted into a 1.5 mL tube containing CTC growth medium and then added to a 12-well ULA plate. Cells were then classified into single cells or clusters and quantified in the well. From the same sort, 100-500 BR16-GFP cells were spiked in 500 µL healthy donor blood and captured on a MyCTC chip. The number of single cells and clusters were then quantified for each chip and well, and the percentage of clusters was determined.

### On-chip culture of CTC-derived cell lines and patient-derived cancer cells from liquid biopsies

GFP-tagged human CTC-derived Brx50 cells or patient-derived cancer cells were added to the capture chamber of the device via a syringe pump at a flow rate of 50 µL min^−1^, and cells were cultured on-chip under hypoxic conditions (5% O_2_). After the isolation of cancer cells from liquid biopsies, e.g., blood or ascites fluid, the chamber was washed with 1 mL 1X PBS. Subsequently, the wash solution was exchanged with CTC growth medium. CTC growth medium (RPMI 1640 Medium (Invitrogen, 52400-025) containing 20 ng mL^−1^ recombinant human epidermal growth factor (Gibco, PHG0313), 20 ng mL^−1^ recombinant human fibroblast growth factor (Gibco, 100-18B), 1X B27 supplement (Invitrogen, 17504-044) and 1X antibiotic-antimycotic (Invitrogen, 15240062)) was added every 48–72 h. A total volume of 100 µL CTC growth medium was added to a truncated 20–200 µL pipette tip, which was inserted into the capture chamber inlet, and an empty tip was inserted into the outlet, creating a steady flow of medium throughout the entire chamber. Generally, while for some patient-derived cancer cells proliferation is indefinite (i.e., we are able to maintain patient-derived cell lines), most proliferation is limited to a few days or weeks. This is sufficient for drug screen purposes but insufficient to derive permanently growing cell lines.

### Microscopy

Patient-derived ascites fluid samples were applied to the MyCTC chip as previously described, and detection of cancerous cells by on-chip live cell immunostaining was carried out using an antibody cocktail of AF-488 anti-EpCAM (Cell Signalling Technologies; CST#5198), AF-488 anti-Her2 (Biolegend; 324410), FITC anti-EGFR (Genetex; GTX11400) and either AF647 or BV605 anti-CD45 (Biolegend; 304042). All brightfield and fluorescence imaging of cells on the chip was carried out using either a Leica DM IL LED or a K5 microscope.

### Drug screen

The drug screen protocol was carried out for the CTC-derived Brx50 cell line and for ascites fluid samples from cancer patients. The following drugs were tested at 5 µM diluted in DMSO: carboplatin, gemcitabine, capecitabine, topotecan, and navelbine. Pure DMSO was used as a control. The samples were transferred to the drug chambers using a syringe pump at 30 µL min^−1^. A total volume of 100 µL medium was added to each chamber using a 20–200 µL tip inserted into the inlet. This medium contained, in addition to the corresponding drug for each chamber, a 1:1000 dilution of the reagent from the Realtime-Glo MT Cell Viability Assay (Promega G9711). Cell viability was recorded via luminescence measurements (IVIS In Vivo Imaging System, Perkin Elmer). Further analysis was carried out using Living Image Software (Perkin Elmer). The relative survival rate was calculated as the ratio of the endpoint luminescence value to the initial luminescence value and normalized to the survival rate value of the control group.

## Results

### Design of the MyCTC chip

**Fig. 1:**
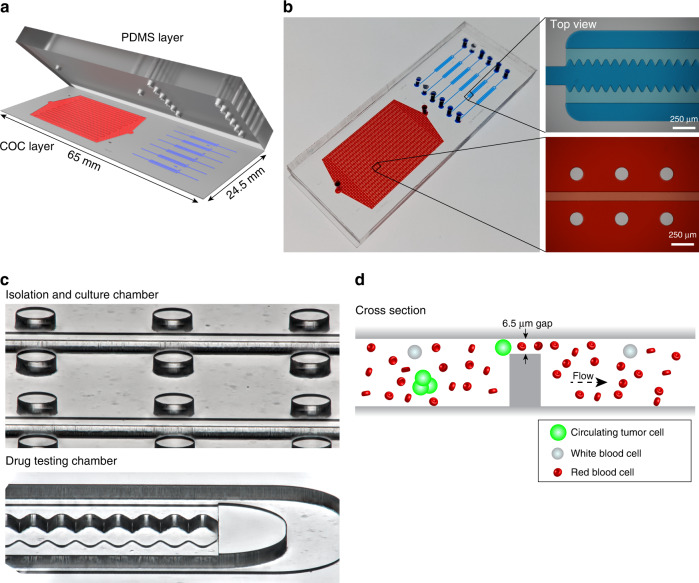
MyCTC chip design. **a** Design of the MyCTC chip, containing a PDMS layer (*top*) and COC layer including the microfluidic structures (*bottom*). **b** Image showing the MyCTC chip, including a detailed view of the capture and culture section (*red*) and drug screen chamber (*blue*). **c** Focus stacked images showing the capture and culture chamber and drug screen chamber. **d** Schematic representation of the CTC capture process of the MyCTC chip

The MyCTC chip was designed to combine in a single device (i) the capture and culture of cancer cells from whole blood, plural effusion fluid, ascites fluid or other body fluids without any preprocessing step and (ii) drug screen of different anticancer agents on cultured cancer cells. The top layer consists of polydimethylsiloxane (PDMS) including an inlet and outlet, facilitating constant gas exchange with the outer atmosphere, whereas the bottom layer containing the microfluidic structures is made of rigid cyclic olefin copolymer (COC), which has a high biocompatibility with primary cells (Fig. 1a and Fig. S1A–S1C). The first section of the MyCTC chip includes a CTC capture and culture chamber (volume of 20 µL) containing longitudinal separation structures with 17 serpentines, and the second section comprises six individual drug screen chambers (volume of 0.4 µL per drug chamber), each composed of a waved opening, allowing entrapment of translocated cells into microwells (Fig. 1b, c). The median height of the MyCTC chip is 45.1 ± 0.7 µm, guiding the cells to a terminal gap of 6.4 ± 0.1 µm. Thus, most blood cells (e.g., WBCs and RBCs) can pass through given their smaller size and higher deformability, and cells characterized by increased size or rigidity—such as cancer cells—are entrapped and enriched (Fig. 1d and Fig. S1D, S1E).

### Characterization of MyCTC chip performance

**Fig. 2:**
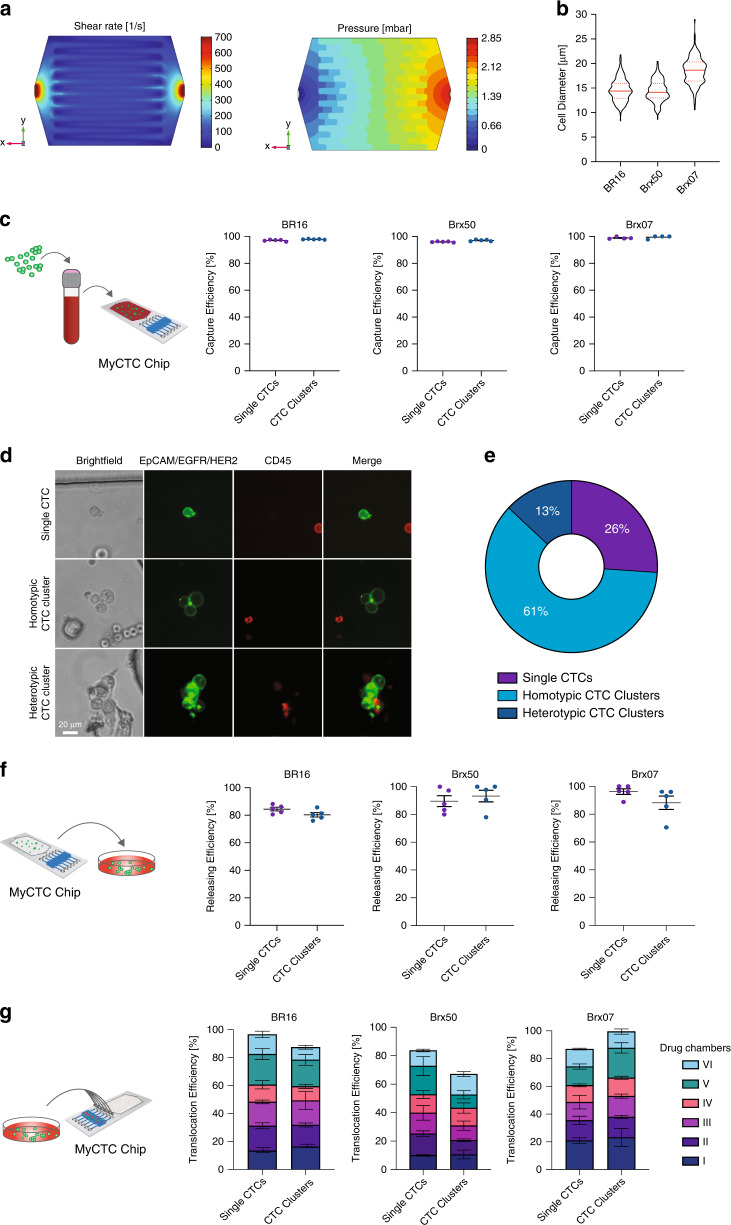
Capture, release and translocation efficiency. **a** Fluidic dynamic simulation showing the distribution of shear rates (*left*) and pressure drop (*right*) within the culture and capture section at a constant flow rate of 50 µL min^−1^. The viscosity of blood was set to 1–3 mPa∙s. **b** Size distribution of CTC-derived cell lines BR16, Brx50 and Brx07. Violin plots show the 25th, 50th and 75th percentiles. **c** Representation of the experimental design (*left*). Dot plots show the capture efficiency of single CTCs and CTC clusters from GFP- or RFP-tagged BR16, Brx50 and Brx07 cells spiked in healthy donor blood (*right*); *n* = 5 for BR16 and Brx50, *n* = 4 for Brx07; error bars represent s.e.m. **d** Representative brightfield and fluorescence images of single CTCs and homotypic and heterotypic CTC clusters isolated from the peripheral blood of a metastatic breast cancer patient using a MyCTC chip. Captured cells were stained with anti-EpCAM/EGFR/HER2 (*green*) and CD45 (*red*) antibodies. **e** Pie chart showing the percentages of single CTCs and homotypic and heterotypic CTC clusters isolated in **d**. **f** Representation of the experimental design (*left*). Dot plot showing the release efficiency from captured single and clustered CTCs of GFP- or RFP-tagged BR16, Brx50 and Brx07 cells (*right*); *n* = 5; error bars represent s.e.m. **g** A representation of the experimental design (*left*). Bar plot showing translocation efficiency from captured single and clustered CTCs of GFP- or RFP-tagged BR16, Brx50 and Brx07 cells into the six drug screening chambers (*right*); *n* = 3; error bars represent s.e.m

To characterize the performance of the MyCTC chip, we first conducted a COMSOL Multiphysics 5.1 simulation to identify an optimal flow rate within the capture and culture section. We reasoned that the flow rate should not lead to shear forces that exceed those experienced by cancer cells within the human circulatory system. The fluid viscosity was set to 1–3 mPa∙s, which represents the estimated viscosity of human blood at room temperature. We found that a flow rate of 50 µL min^−1^ resulted in a maximal shear rate of 100 s^−1^ (translating into 0.2–0.6 N m^−2^) at the filter section and a maximal pressure drop of 2.85 mbar within the capture and culture chamber, not exceeding critical shear stress levels for mammalian cells (Fig. 2a)^50,51^. Based on this simulation, various flow rates for CTC capture on the MyCTC chip were tested, showing that a capture flow rate of 50 µL min^−1^ results in a higher capture efficiency for CTC clusters using different CTC-derived cell lines (Fig. S2A). Next, we sought to identify the capture efficiency of the MyCTC chip by processing healthy donor blood spiked with three different GFP- or RFP-tagged human CTC-derived cell lines, each characterized by varying mean cell diameters (14.38 ± 2.29 µm for GFP-tagged BR16 cells; 14.16 ± 2.16 µm for GFP-tagged Brx50 cells; and 18.63 ± 2.67 µm for RFP-tagged Brx07 cells) and thus highly representative of freshly isolated CTCs of patients (Fig. 2b). First, cells were resuspended, resulting in single CTCs and CTC clusters (ranging from 2–20 cells), and subsequently, 500 cells were spiked into 1 mL healthy donor blood and processed through the MyCTC chip at a flow rate of 50 µL min^−1^. The MyCTC chip captured single CTCs with a mean capture efficiency percentage of 97.02% ± 0.60 (BR16), 95.99% ± 0.36 (Brx50), and 98.89% ± 0.75 (Brx07); CTC clusters were captured with a mean capture efficiency of 97.87% ± 0.24 (BR16), 97.08% ± 0.60 (Brx50), and 99.42% ± 1.17 (Brx07) (Fig. 2c). To test the MyCTC chip in a more clinically relevant setting, we applied a whole blood sample from a patient with metastatic breast cancer (Table S1) and stained the captured cells on-chip with antibodies against EPCAM, EGFR and HER2 to identify cancer cells and with antibodies against CD45 to discriminate the remaining haematopoietic cells. The MyCTC chip successfully captured not only single CTCs but also homotypic and heterotypic clusters, providing a comprehensive spectrum of CTC types (Fig. 2d). In total, 6 single CTCs (26%), 14 homotypic CTC clusters (61%) and 3 heterotypic CTC clusters (13%) were detected in 18.5 mL whole blood (Fig. 2e). Next, we determined the release efficiency of captured single CTCs and CTC clusters, i.e., the ability to extract captured CTCs into a cell suspension for possible downstream analyses. With an inverted flow rate of 30 µL s^−1^, the MyCTC chip showed a mean release percentage of 84.49% ± 3.05 (BR16), 89.62% ± 3.89 (Brx50), and 96.26% ± 2.05 (Brx07) for single CTCs and 80.36% ± 3.64 (BR16), 93.28% ± 4.19 (Brx50), and 88.24% ± 4.82 (Brx07) for CTC clusters (Fig. 2f). Subsequently, captured single CTCs and CTC clusters were translocated into the six drug screen chambers with a mean efficiency of 96.60% ± 3.39 (BR16), 83.68% ± 13.53 (Brx50), and 86.89% ± 4.51 (Brx07) for single CTCs and 87.47% ± 2.84 (BR16), 67.11% ± 4.89 (Brx50), and 99.59% ± 0.4 (Brx07) for CTC clusters (Fig. 2g), demonstrating even distribution for subsequent drug testing. We then determined the capture purity on-chip, showing mean CTC/WBC ratios of 0.504 ± 0.21 (BR16), 0.978 ± 0.115 (Brx50) and 1.015 ± 0.268 (Brx07); mean postrelease CTC/WBC ratios were found to be 0.599 ± 0.086 (BR16), 0.928 ± 0.332 (Brx50) and 1.179 ± 0.285 (Brx07) (Fig. S2B). We next sought to quantify cell viability on the chip and postrelease by counting both the live and dead cells. The mean percentages of viable cells during standard CTC culture were 79.81% ± 2.269 (BR16), 68.7% ± 5.4 (Brx50) and 82.89% ± 4.78 (Brx07). Upon capture via the MyCTC chip, the viability showed mean values of 88.87% ± 5.8 (BR16), 70.1% ± 9.74 (Brx50), and 95.62% ± 2.68 (Brx07) on-chip and mean values of 86.83% ± 3.91 (BR16), 66.43% ± 12.86 (Brx50), and 88.64% ± 4.1 (Brx07) postrelease (Fig. S2C). Finally, we quantified the extent of artificial clustering of CTCs upon capture in the MyCTC chip. To this end, we sorted a single cell suspension of BR16 cells and quantified the percentage of CTC clusters in static conditions (immediately after sorting) as well as upon capture on the MyCTC chip, showing no significant difference between the two conditions (Fig. S2D). Together, these experiments demonstrate a high capture efficiency of the MyCTC chip, along with the ability to successfully release captured cancer cells in a viable state and allocate them in six drug screen chambers.

### On-chip culture and drug screen of patient-derived cancer cells from liquid biopsies

**Fig. 3:**
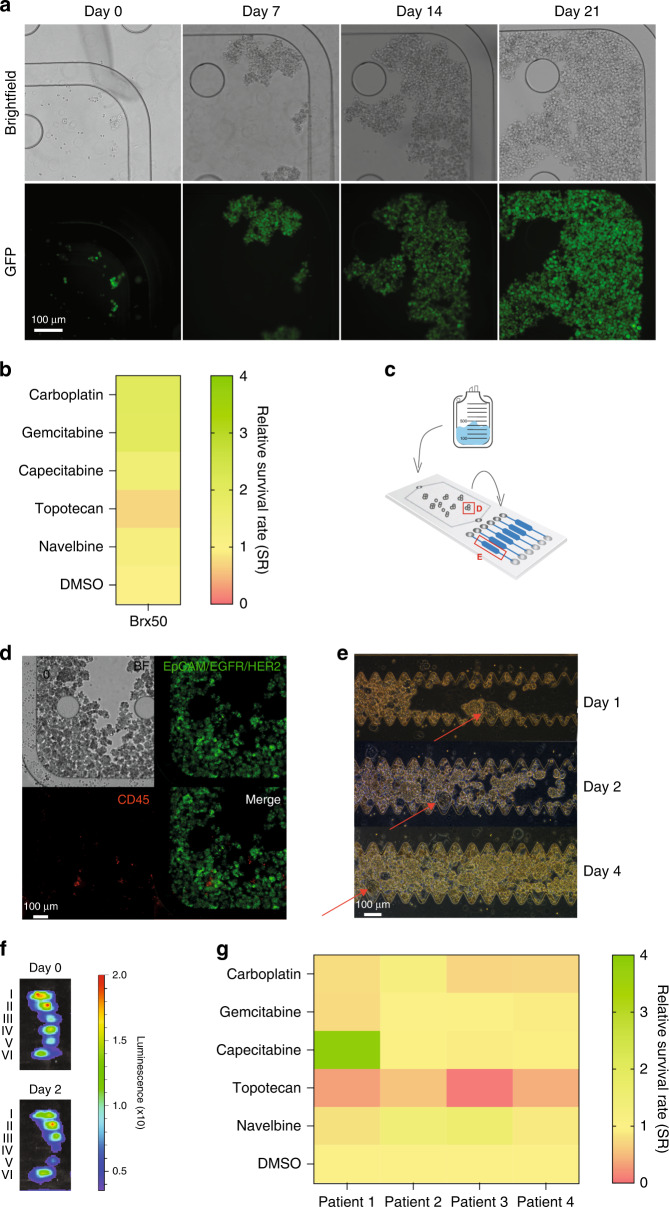
MyCTC chip culture and drug screening. **a** Brightfield and fluorescence images at different time points (days 0, 7, 14, 21) showing the growth of the GFP-tagged Brx50 CTC line inside the culture chamber of the MyCTC chip. **b** Heatmap representing the average relative survival rate (*n* = 2) of Brx50 cells at the endpoint measurement after two days of chemotherapeutic agent (I–VI) treatment**. c** Schematic representation of the workflow for patient-derived ascites fluid processing with the MyCTC chip. Red squares (**d**, **e**) represent the position on the chip that was used for imaging cell growth shown in **d**, **e**. **d** Representative brightfield and immunofluorescence images of captured patient-derived ascites fluid cancer cells in the capture and culture chamber stained for EpCAM/EGFR/HER2 (*green*) and CD45 (*red*). **e** Representative brightfield images of patient-derived ascites fluid cells in the drug screen chambers at different time points after translocation (days 1, 2, 4). *Red* arrows indicate the imaging reference point. **f** Representative images showing the bioluminescence signal of the drug screen chambers containing cancer cells from patient-derived ascites fluid samples (Table S1; Patient four) treated with (VI) carboplatin, (V) gemcitabine, (IV) capecitabine, (III) topotecan, (II) navelbine and (I) DMSO control before (day 0) and 2 days after drug treatment. Bioluminescence levels indicate the viability of cancer cells from patient-derived ascites fluid samples. **g** Heatmap representing the average relative survival rate (*n* = 2) of cancer cells from patient-derived ascites fluid at the endpoint measurement

To test whether CTCs can be cultured on the MyCTC chip, we applied 1200 GFP-tagged Brx50 cells, which were captured at a flow rate of 50 µL min^−1^, for subsequent maintenance and expansion. We exchanged the CTC growth medium every second or third day, resulting in successful culture of Brx50 cells within the culture chamber (Fig. 3a, Fig. S2E, F). Next, we sought to test drug susceptibility in CTCs using the MyCTC chip. To this end, we translocated Brx50 cells to the six drug screen chambers at a flow rate of 30 µL min^−1^ and exposed them to a series of chemotherapeutic agents that are frequently used in the clinical setting to treat breast, endometrial, tubal and ovarian cancer patients of different stages. We added media containing 5 µM carboplatin, gemcitabine, capecitabine, topotecan or navelbine diluted in DMSO, along with DMSO control and in combination with a luminescence-based cell viability solution (see Materials and Methods), individually to each drug screen chamber. Once the cells and supplemented media had been added to the chambers, we incubated them for 1 h at 37 °C and measured cell viability (initial luminescence value; Day 0). Subsequently, we cultured the cells under treatment or DMSO control for an additional 48 h before recording the endpoint cell viability (luminescence value; Day 2) and calculated the average relative survival rate (SR) of cells in each drug chamber. We found that cells treated with topotecan showed the strongest decrease in viability compared to the DMSO control (SR = 0.71), suggesting susceptibility of Brx50 cells to topotecan but not to other drugs that were tested (Fig. 3b). Furthermore, to test MyCTC chip drug screen capabilities in a clinical setting, we collected ascites fluid cancer cells from endometrial, tubal and ovarian cancer patients before treatment (Table S1, Fig. S3A and Fig. S3B) and processed them via the MyCTC chip (Fig. 3c). We successfully isolated patient-derived cancer cells within the capture chamber (Fig. 3d) and then transferred them to the drug screen chambers, where they reached confluency at Day 4 (Fig. 3e and Fig. S2G). For the drug screen, we treated the patient-derived cells with the same chemotherapeutic agents that were tested on Brx50 cells, as described above. The endpoint cell viability was measured 52–54 h post-drug treatment (Fig. 3f), and the SR values were calculated, comparing each drug to the nontreated DMSO control (Fig. 3g). We observed a viability decrease with topotecan for all patient samples, with an average SR of 0.32 (*patient 1*), 0.56 (*patient 2*), 0.08 (*patient 3*), and 0.41 (*patient 4*), suggesting that topotecan had the greatest effect on cancer cell viability. Additionally, cancer cells from patient two were not noticeably susceptible to any drug other than topotecan (SR ≥ 1), while cancer cells from the other patients were additionally susceptible to carboplatin, with an average SR of 0.76 (*patient 1*), 0.70 (*patient 3*), and 0.72 (*patient 4*). Moreover, cancer cells from patient one were also susceptible to gemcitabine (SR = 0.74). These data provide proof-of-concept evidence for using MyCTC chips for the isolation, cultivation and drug screen of patient-derived cancer cells to guide treatment decisions in advanced cancer patients.

## Discussion

Here, we introduce a microfluidic device that enables the isolation, cultivation and drug screen of primary cancer cells from unprocessed liquid biopsies of patients with cancer, with the purpose of enhancing liquid biopsy-based personalized medicine approaches. The antigen-independent dynamic capture of single, homotypic and heterotypic clusters of cancer cells using low flow rates and subsequent culture provides combined capabilities that are insufficiently achieved with other technologies. Existing approaches for primary cancer cell isolation and cultivation from liquid biopsies mostly involve preprocessing steps, which may not only lead to cell loss but can also interfere with culture success. In this context, we identified an optimal flow rate resulting in efficient isolation of viable single and clustered cancer cells, allowing us to successfully expand them and use them for drug susceptibility assays.

Currently, the clinical application of CTCs most frequently involves their enumeration and molecular phenotyping. However, a more comprehensive characterization of CTCs investigating their susceptibility to anticancer drugs in a short time is crucial to implement the concept of personalized medicine for treatment decisions. During tumour evolution, genomic instability contributes to the emergence of resistant tumour subclones, leading to highly complex genetic landscapes, especially in advanced disease stages, concurring with the inefficacy of anticancer treatments. After failure of multiple lines of therapy and tumour progression, especially in cases of tumour resistance, treatment decision guidelines are often insufficient. In contrast, our microfluidic method provides the possibility of direct small-scale drug screens with patient-derived cancer cells from liquid biopsies at virtually any disease stage. The combination of easy handling, low manufacturing costs of thermoplastics, expansion of cancer cells on-chip and the possibility of testing multiple drugs individually at the same time eliminates many challenges that previously hindered the translation of such technologies into the clinic. Future prospective studies will be needed to demonstrate the suitability of this technology for clinical decision-making in advanced cancer settings. In the long term, we envision that the “try and see” attitude that characterizes the treatment of very advanced cancers might be replaced with a more personalized, liquid biopsy-based approach for the identification of specific vulnerabilities.

## Supplementary information

Supplemental Material

## References

[1] Sung H (2021). Global Cancer Statistics 2020: GLOBOCAN estimates of incidence and mortality worldwide for 36 cancers in 185 countries. CA Cancer J. Clin..

[2] Chaffer CL, Weinberg RA (2011). A perspective on cancer cell metastasis. Science.

[3] Rosenkrantz AB (2015). Clinical utility of quantitative imaging. Acad. Radio..

[4] Dowlati A (2001). Sequential tumor biopsies in early phase clinical trials of anticancer agents for pharmacodynamic evaluation. Clin. Cancer Res.

[5] Chen PL (2016). Analysis of immune signatures in longitudinal tumor samples yields insight into biomarkers of response and mechanisms of resistance to immune checkpoint blockade. Cancer Disco..

[6] Mader S, Pantel K (2017). Liquid biopsy: current status and future perspectives. Oncol. Res Treat..

[7] Zhu Z, Qiu S, Shao K, Hou Y (2018). Progress and challenges of sequencing and analyzing circulating tumor cells. Cell Biol. Toxicol..

[8] McGranahan N, Swanton C (2017). Clonal heterogeneity and tumor evolution: past, present, and the future. Cell.

[9] Gennari A (2021). ESMO Clinical Practice Guideline for the diagnosis, staging and treatment of patients with metastatic breast cancer. Ann. Oncol..

[10] Kilgour E, Rothwell DG, Brady G, Dive C (2020). Liquid biopsy-based biomarkers of treatment response and resistance. Cancer Cell.

[11] Ignatiadis M, Sledge GW, Jeffrey SS (2021). Liquid biopsy enters the clinic—implementation issues and future challenges. Nat. Rev. Clin. Oncol..

[12] Belotti Y, Lim CT (2021). Microfluidics for liquid biopsies: recent advances, current challenges, and future directions. Anal. Chem..

[13] Pantel K, Brakenhoff RH, Brandt B (2008). Detection, clinical relevance and specific biological properties of disseminating tumour cells. Nat. Rev. Cancer.

[14] Follain G (2020). Fluids and their mechanics in tumour transit: shaping metastasis. Nat. Rev. Cancer.

[15] Pantel K, Speicher MR (2016). The biology of circulating tumor cells. Oncogene.

[16] Bidard FC (2021). Efficacy of circulating tumor cell count-driven vs clinician-driven first-line therapy choice in hormone receptor-positive, ERBB2-negative metastatic breast cancer: the STIC CTC randomized clinical trial. JAMA Oncol..

[17] Lorente D (2018). Circulating tumour cell increase as a biomarker of disease progression in metastatic castration-resistant prostate cancer patients with low baseline CTC counts. Ann. Oncol..

[18] Goldkorn A (2014). Circulating tumor cell counts are prognostic of overall survival in SWOG S0421: a phase III trial of docetaxel with or without atrasentan for metastatic castration-resistant prostate cancer. J. Clin. Oncol..

[19] Arrazubi V (2019). Circulating tumor cells in patients undergoing resection of colorectal cancer liver metastases. Clinical utility for long-term outcome: a prospective trial. Ann. Surg. Oncol..

[20] Theodoropoulos PA (2010). Circulating tumor cells with a putative stem cell phenotype in peripheral blood of patients with breast cancer. Cancer Lett..

[21] Baccelli I (2013). Identification of a population of blood circulating tumor cells from breast cancer patients that initiates metastasis in a xenograft assay. Nat. Biotechnol..

[22] Papadaki MA (2014). Co-expression of putative stemness and epithelial-to-mesenchymal transition markers on single circulating tumour cells from patients with early and metastatic breast cancer. BMC Cancer.

[23] Powell AA (2012). Single cell profiling of circulating tumor cells: transcriptional heterogeneity and diversity from breast cancer cell lines. PLoS One.

[24] Aceto N (2014). Circulating tumor cell clusters are oligoclonal precursors of breast cancer metastasis. Cell.

[25] Castro-Giner F, Scheidmann MC, Aceto N (2018). Beyond enumeration: functional and computational analysis of circulating tumor cells to investigate cancer metastasis. Front Med (Lausanne).

[26] Aceto N (2020). Bring along your friends: homotypic and heterotypic circulating tumor cell clustering to accelerate metastasis. Biomed. J..

[27] Chemi F (2020). Publisher correction: pulmonary venous circulating tumor cell dissemination before tumor resection and disease relapse. Nat. Med.

[28] Izar B (2020). A single-cell landscape of high-grade serous ovarian cancer. Nat. Med.

[29] Hodge C, Badgwell BD (2019). Palliation of malignant ascites. J. Surg. Oncol..

[30] Yu M (2014). Cancer therapy. Ex vivo culture of circulating breast tumor cells for individualized testing of drug susceptibility. Science.

[31] Soler A (2018). Autologous cell lines from circulating colon cancer cells captured from sequential liquid biopsies as model to study therapy-driven tumor changes. Sci. Rep..

[32] Hamilton G, Burghuber O, Zeillinger R (2015). Circulating tumor cells in small cell lung cancer: ex vivo expansion. Lung.

[33] Kar R (2017). Establishment of primary cell culture from ascitic fluid and solid tumor obtained from epithelial ovarian carcinoma patients. Int J. Gynecol. Cancer.

[34] Liu W (2017). Circulating tumor cells in prostate cancer: precision diagnosis and therapy. Oncol. Lett..

[35] Khoo BL (2018). Expansion of patient-derived circulating tumor cells from liquid biopsies using a CTC microfluidic culture device. Nat. Protoc..

[36] Sharma S (2018). Circulating tumor cell isolation, culture, and downstream molecular analysis. Biotechnol. Adv..

[37] Riethdorf S (2007). Detection of circulating tumor cells in peripheral blood of patients with metastatic breast cancer: a validation study of the CellSearch system. Clin. Cancer Res.

[38] Lu NN (2015). Biotin-triggered decomposable immunomagnetic beads for capture and release of circulating tumor cells. ACS Appl Mater. Interfaces.

[39] Yoo CE (2016). Vertical magnetic separation of circulating tumor cells for somatic genomic-alteration analysis in lung cancer patients. Sci. Rep..

[40] Armbrecht L (2020). Quantification of protein secretion from circulating tumor cells in microfluidic chambers. Adv. Sci. (Weinh.).

[41] Sarioglu AF (2015). A microfluidic device for label-free, physical capture of circulating tumor cell clusters. Nat. Methods.

[42] Edd JF (2020). Microfluidic concentration and separation of circulating tumor cell clusters from large blood volumes. Lab Chip.

[43] Sollier E (2014). Size-selective collection of circulating tumor cells using Vortex technology. Lab Chip.

[44] Zhu Z (2022). High-throughput and label-free enrichment of malignant tumor cells and clusters from pleural and peritoneal effusions using inertial microfluidics. Lab Chip.

[45] Hou HW (2013). Isolation and retrieval of circulating tumor cells using centrifugal forces. Sci. Rep..

[46] Miller MC, Robinson PS, Wagner C, O’Shannessy DJ (2018). The parsortix cell separation system-A versatile liquid biopsy platform. Cytom. A.

[47] Crawford J, Dale DC, Lyman GH (2004). Chemotherapy-induced neutropenia: risks, consequences, and new directions for its management. Cancer.

[48] Zheng LM, Zychlinsky A, Liu CC, Ojcius DM, Young JD (1991). Extracellular ATP as a trigger for apoptosis or programmed cell death. J. Cell Biol..

[49] Salvestrini V (2017). Extracellular ATP induces apoptosis through P2X7R activation in acute myeloid leukemia cells but not in normal hematopoietic stem cells. Oncotarget.

[50] Ludwig A, Kretzmer G, Schugerl K (1992). Determination of a “critical shear stress level” applied to adherent mammalian cells. Enzym. Micro. Technol..

[51] Shive MS, Salloum ML, Anderson JM (2000). Shear stress-induced apoptosis of adherent neutrophils: a mechanism for persistence of cardiovascular device infections. Proc. Natl Acad. Sci. USA.

