# Supplementary material for: MyCTC chip: microfluidic-based drug screen with patient-derived tumour cells from liquid biopsies
Source: Microsyst Nanoeng. 2022 Dec 20;8:130. doi: 10.1038/s41378-022-00467-y (PMC9763115; doi:10.1038/s41378-022-00467-y)
Supplement: Supplementary file 1 — Supplemental Material [file 41378_2022_467_MOESM1_ESM.docx]

**Supplementary information**


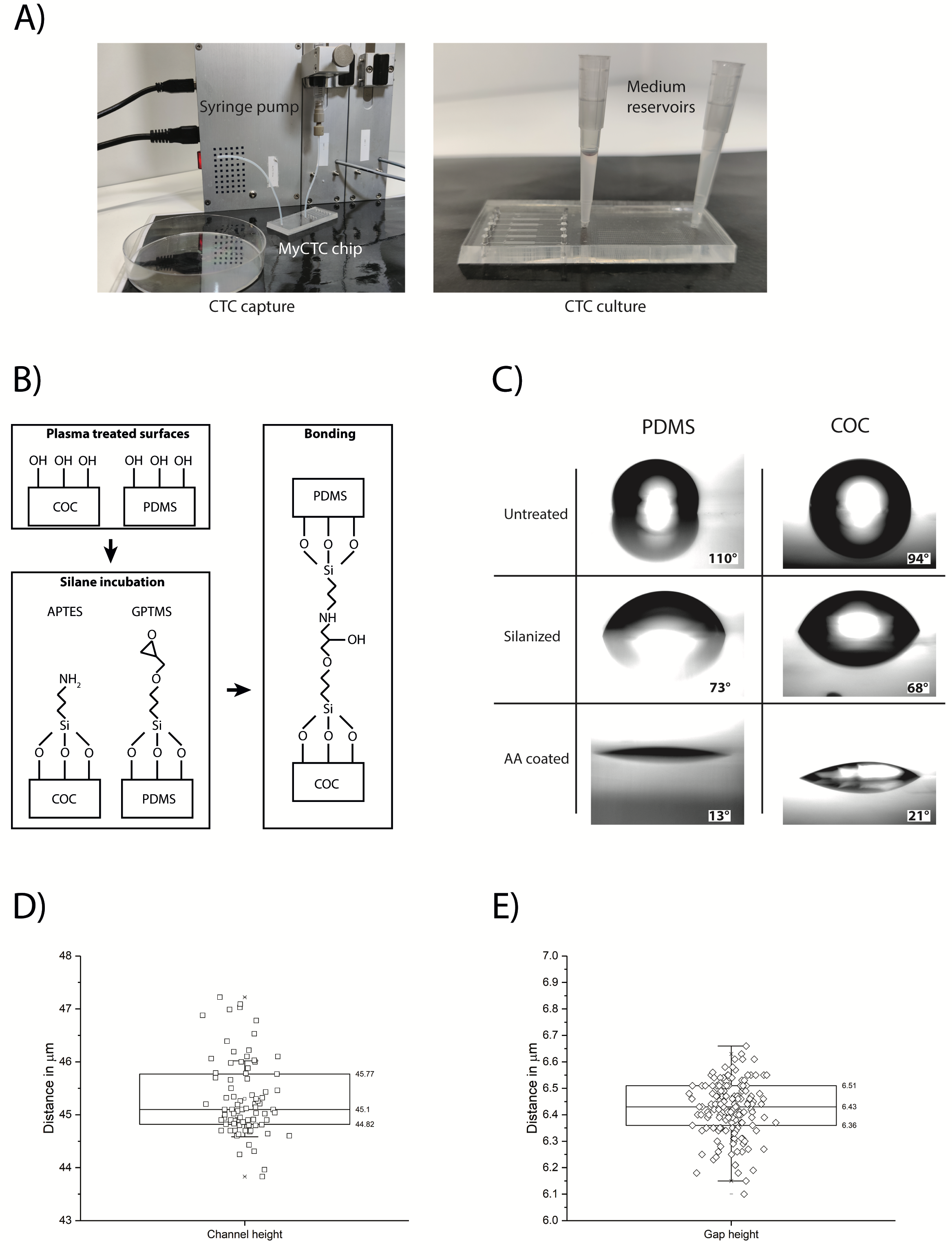


**Figure S1**: **Biophysical/chemical properties and setup of the MyCTC chip.** **A)** Images showing the MyCTC chip connected to the syringe pump (*left*) and to media reservoirs (*right*). **B)** Schematic of the surface chemistry employed for the covalent bonding of PDMS to COC. **C)** Contact angle measurements of DI water on untreated, silanized and anti-adherence (AA) solution-coated PDMS and COC samples indicating successful surface modification. **D)** Median height of the imprinted channel structures on COC. The box plot shows the 25^th^ and 75^th^ percentiles and median. **E)** Median gap height of the imprinted channel structures on COC. The box plot shows the 25^th^ and 75^th^ percentiles and median.


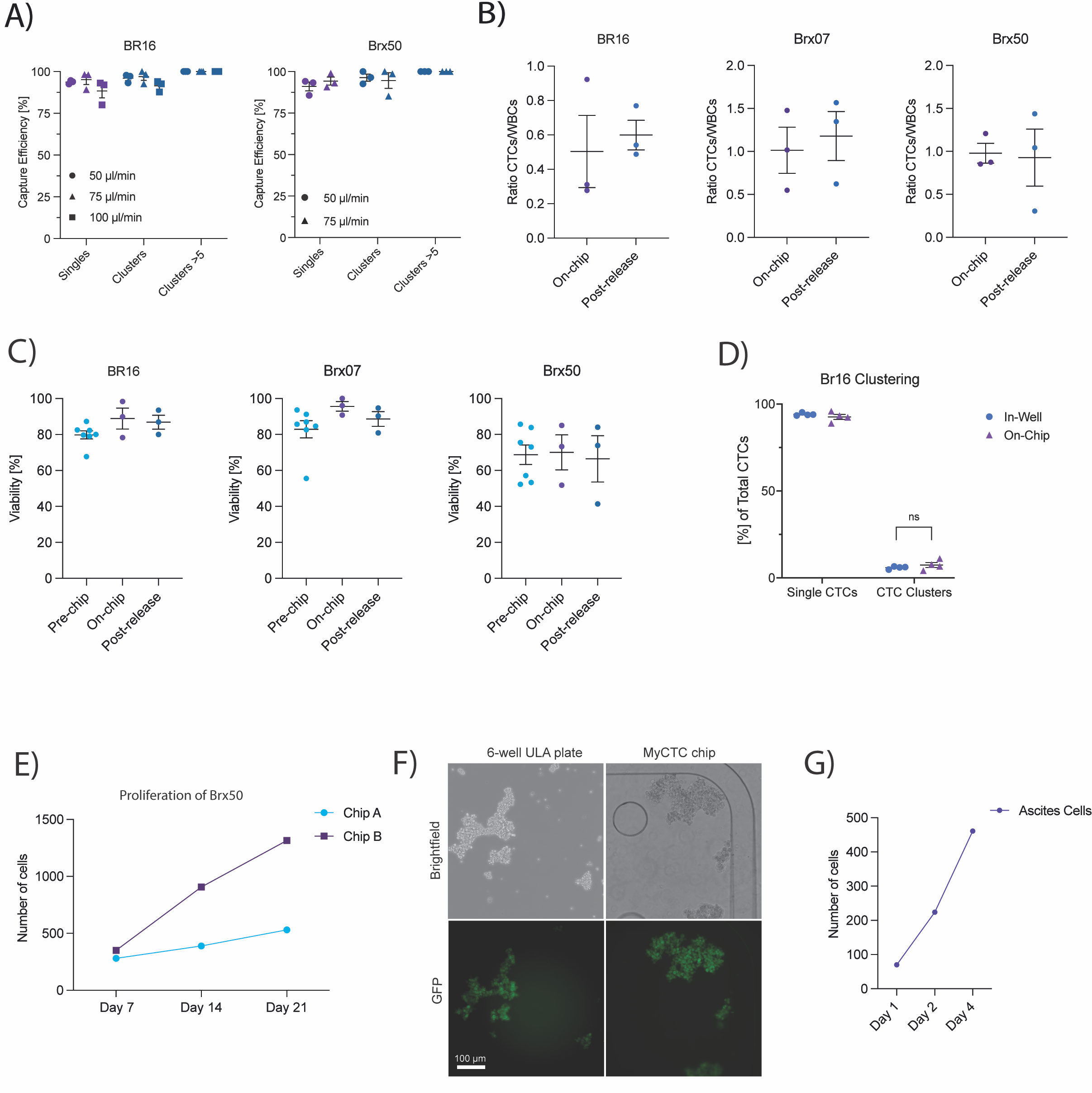


**Figure S2: Flow rate, purity and CTC viability assessment of the MyCTC Chip.**

**A)** Dot plots showing the capture efficiency from captured single and clustered CTCs of GFP-tagged BR16 cells (*left*) or Brx50 cells (*right*) with a flow rate of 50 µL min^-1^ (*dot*), 75 µL min^-1^ (*triangle*) or 100 µL min^-1^ (*square*); *n* = 3; error bars represent s.e.m. **B)** Dot plot showing purity (ratio CTCs/WBCs) of GFP- or RFP-tagged BR16, Brx07 or Brx50 cells on-chip and postrelease; *n* = 3; error bars represent s.e.m **C)** Dot plot showing viability of GFP- or RFP-tagged BR16, Brx07 or Brx50 cells prechip (*n=*7*)*, on-chip and postrelease (*n* = 3); error bars represent s.e.m. **D**) Post-sorting percentages of single and cluster CTCs in a 12-well plate and upon capture with the MyCTC chip; *n* = 4; error bars represent s.e.m; Mann‒Whitney-U test (ns = not significant). **E**) Growth curves of GFP-tagged Brx50 cells in the culture chamber of the MyCTC chip; *n* = 2. **F**) Representative brightfield and fluorescence images of GFP-tagged Brx50 cells after one week in culture in a 6-well plate or within the culture chamber of the MyCTC chip. **G**) Growth curve of patient-derived ascites fluid cells in the drug screen chamber of the MyCTC chip.


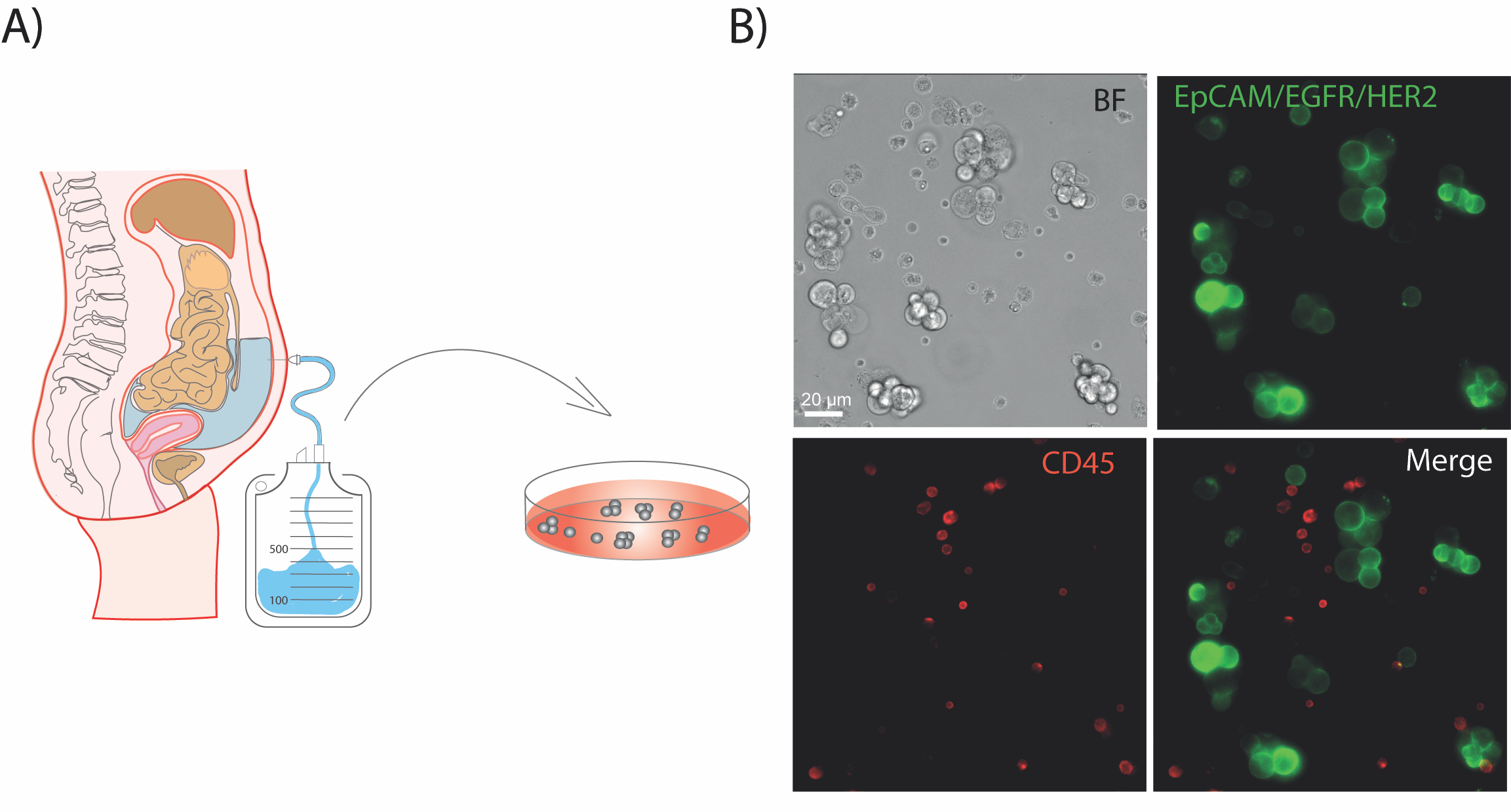


**Figure S3**: **Schematic of ascites collection and immunostained cancer cells after isolation.** **A)** Schematic of an abdominal tap to collect ascites fluid samples and live immunostaining. **B)** Representative brightfield and fluorescence images of patient-derived ascites fluid samples stained with EpCAM/EGFR/HER2 (*green*) and CD45 (*red*) confirming the presence of cancer cells.

**Table S1**: **Clinical features of five cancer patients (with endometrial, tubular, ovarian or breast cancer) with detectable circulating tumour cells (CTCs) or cancer cells in ascites fluid samples.** The table shows the sample type that was used from these patients, age (in years) at the moment of primary diagnosis, cancer type, stage (based on the Federation of Gynaecology and Obstetrics (FIGO) uterine cervical cancer staging system and TNM), percentage of ER+ and PR+ cells, microsatellite instability, TP53 status, L1CAM status and metastasis status.
